# Storytelling to communicate public health messages during the COVID-19 pandemic; A systematic review: Priyamvada Paudyal

**DOI:** 10.1093/eurpub/ckac129.636

**Published:** 2022-10-25

**Authors:** P Paudyal, S Ozkosar

**Affiliations:** Primary Care and Public Health, Brighton and Sussex Medical School, Brighton, UK

## Abstract

**Background:**

Storytelling has been increasingly used in public health to disseminate health messages in a format that is more understandable and engaging. This project aims to summarize the findings from the studies that used storytelling approach for conveying health messages during the COVID-19 pandemic.

**Methods:**

The protocol for this systematic review is registered in PROSPERO (CRD42021281957). Studies were identified via electronic databases; EMBASE, Ovid MEDLINE and EBSCO using variations of the search terms ‘COVID-19’ and ‘storytelling’. Studies that used storytelling interventions in COVID-19 and published in English after 2020 were eligible for inclusion. Data from eligible full text article was collated in a summary table. Quality of included studies were assessed using Joanna Briggs Institute Checklists.

**Results:**

Of the 4562 studies identified in the electronic search, 11 were eligible for inclusion. Participant numbers in each study varied from 14 to 3746. Eight of the 11 studies included were set online, looking at virtual forms of storytelling. This included online surveys and social media campaigns within hospitals, and online meetings with patients at home. Overall, studies showed that storytelling intervention increased engagement in health communication and has the potential to be used as an effective public health communication tool, for changing health behaviours. Studies were small, six out of 11 were of poor quality.

**Conclusions:**

Storytelling is a widely accepted intervention and has the potential to positively impact communication, empathy and health behaviour. As there is no standardized definition of storytelling, and studies into this area are new, it is important to continually re-evaluate data to enhance understanding and provide standardized guidelines for storytelling in the COVID-19 context.

**Key messages:**

• Storytelling has been increasingly used in public health to disseminate health messages.

• Storytelling has the potential to positively impact communication, empathy and health behaviour.

